# Microplastic Transport and Accumulation in Rural Waterbodies: Insights from a Small Catchment in East China

**DOI:** 10.3390/toxics12100761

**Published:** 2024-10-19

**Authors:** Tom Lotz, Wenjun Chen, Shoubao Su

**Affiliations:** 1 School of Computer Engineering, Jinling Institute of Technology, Hongjing Avenue 99, Nanjing 211169, China; showbo@jit.edu.cn; 2 Jiangsu Key Laboratory of Data Science & Smart Software, Jinling Institute of Technology, Hongjing Avenue 99, Nanjing 211169, China; 3 School of Software Engineering, Jinling Institute of Technology, Hongjing Avenue 99, Nanjing 211169, China; chenwenjun@jit.edu.cn; 4 Key Laboratory of Watershed Geographic Science, Nanjing Institute of Geography and Limnology, Chinese Academy of Sciences, Nanjing 210008, China; 5 School of Computer, Jiangsu University of Science and Technology, Changhui Road 666, Zhenjiang 212003, China

**Keywords:** microplastics, drainage ditches, ponds, environmental pollution, microplastic shape parameters

## Abstract

Microplastic (MP) pollution in agricultural ecosystems is an emerging environmental concern, with limited knowledge of its transport and accumulation in rural waterbodies. This study investigates the distribution and sources of MP in drainage ditches influenced by pond connectivity, land use, and soil properties within a small catchment in Nanjing, East China. Sediment was collected from ditches in 18 sites across forest, agricultural, horticultural, and urban areas. Using laser-directed infrared spectroscopy (LDIR), 922 MP particles were identified. Six materials were dominant: fluororubber (FR), polyethylene terephthalate (PET), polyurethane (PU), acrylonitrile (ACR), chlorinated polyethylene (CPE), and polyethylene (PE). MP concentrations varied by land use and pond connectivity, with ditches above ponds exhibiting higher counts (1700 particles/kg) than those below (1050 particles/kg), indicating that ponds act as MP sinks. The analysis revealed site-specific MP sources, with FR linked to road runoff and PET associated with agricultural practices. Correlations between MP shape and soil properties showed that more compact and filled shapes were more commonly associated with coarser soils. PE particle size was negatively correlated with organic matter. This study highlights the need for targeted strategies to reduce MP pollution in rural landscapes, such as reducing plastic use, ditch maintenance, and improved road runoff management.

## 1. Introduction

The presence of microplastics (MP), i.e., plastic particles of a size below 5 mm, in the environment has gained significant attention, particularly in the last decade [1,2]. MP pollution poses various environmental risks. MP particles have been shown to be ingested by marine and freshwater fish, invertebrates, and zooplankton, which leads to them consuming less natural prey, with negative effects on their growth, reproduction, and survival [3,4,5]. Similar effects have been observed in terrestrial habitats, where MP pollution negatively affected springtails, nematodes, and earthworms [6,7]. Negative effects on plants and photosynthetic organisms have been found in aquatic and terrestrial environments [8,9]. Through ingestion by plants and animals, MP particles move up the food chain and can eventually be consumed by humans. Other human exposure modes to MP pollution are food, bottled water, and even air [10]. MP has been discovered in various human body tissues and may cause adverse health effects from inflammatory reactions and disturbed gut microbiota to autoimmune reactions [11,12]. Furthermore, MP particles interact with other pollutants in water and soil [13,14] and can alter bacterial communities, potentially affecting antibiotic resistance in human and animal pathogens [15,16].

Many human activities introduce MP into the environment from both point sources, including industrial production and waste disposal, and non-point sources, ranging from cosmetics, fabrics, and agriculture to road traffic. The specific combination of MP sources depends on the respective environment [17,18]. The type of MP (material and shape) allows for the estimation of the source of the particles [19]. MP transport and accumulation are two closely related processes that dynamically interact with each other [20,21,22]. Depending on size and material, MP transport from the source occurs through different vectors. Small MP particles can be transported by wind [23] and are dispersed in the soil by biota [24]. Most MP particles are carried by moving water, either mixed with sediments or floating in the water [25,26]. To tackle the environmental and health-related issues caused by MP, it is necessary to better understand MP sources and transport and accumulation processes [27].

In rural areas, agriculture is a significant source of MP in terrestrial and aquatic ecosystems [28]. MP is introduced into the environment through agricultural practices, including the use of biosolids, mulching films, and other agricultural plastics, which are often left to decay in the soil [29,30]. Despite recent progress in investigating MP in different environments, the behavior of MP in agricultural systems remains poorly understood [31,32]. The amount and mobility of MP in agricultural fields depend on a series of factors, including MP properties, soil properties, and farming activities [33]. While research has shed light on these individual contributors to MP presence in the agricultural context [34,35], the comprehensive understanding of how these factors interact remains limited. Current studies often isolate variables, neglecting the complex, interconnected dynamics that characterize agricultural ecosystems. MP transport in agricultural fields occurs along with eroded soil in surface runoff, wind transport, and soil biota [36], leading to their subsequent movement into ditches and ponds, primarily via surface runoff [17,37].

Rural ponds and drainage ditches are two widespread types of waterbodies in the agricultural areas of East China. Rural ponds are used for water storage and low-intensity food production and are often vital to everyday countryside life, local hydrology, and agriculture [38]. The quality of rural pond water has been directly linked to the source and volume of inflowing water, the effects of the surrounding landscape [39], and agricultural land cover [40]. MP in the water and sediment of ponds may harm populations using the water for irrigation or consuming food grown in the ponds [41], highlighting the need to improve the understanding of MP transport and accumulation processes [27]. Previous MP studies in ponds mainly focused on those used for crab and fish farming [42,43]. However, very little is known about MP in the much more common rural ponds, leading to shortcomings in understanding the role of these ponds in the transport and accumulation processes of MP. Drainage ditches have been shown to serve as pathways for transporting phosphorus, nitrogen, herbicides, and heavy metals [44,45]. Previous studies linked pollutant transport in ditches to management practices, catchment properties, and hydrological connectivity [46,47,48,49]. This study investigates the distribution and sources of MPs within a small agricultural catchment in East China, examining how connectivity with rural ponds and factors such as land cover, soil properties, and agricultural activities influence MP concentrations in ditch sediments. By integrating these factors, the study aims to provide a more comprehensive understanding of MP transport and accumulation in rural waterbodies, addressing the knowledge gap regarding the interactions between ditches, rural ponds, and the surrounding landscape in MP pollution dynamics.

## 2. Materials and Methods

### 2.1. Study Site

**Figure 1:**
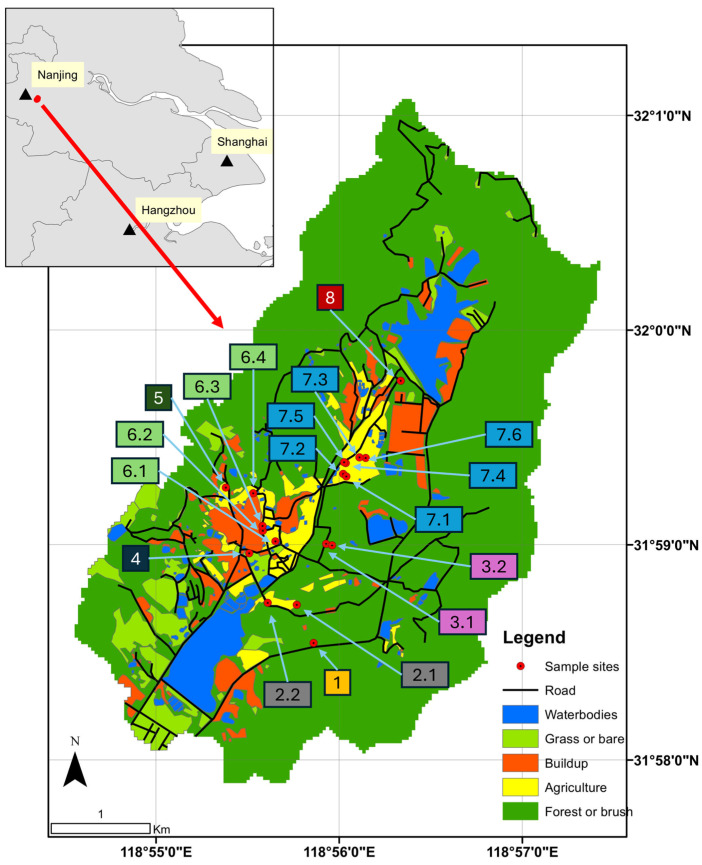
A land cover map of the research area, the sample sites, and an indication of its position near Nanjing. Label color indicates the different sampling complexes.

The research catchment is situated east of Nanjing, in Jiangsu province, China (Figure 1). It is part of the western end of the Tangshan Mountain area. The catchment covers an area of 20.3 km^2^, of which 76% is forest, 7% is water, 7% is built-up (urban and paved areas), 5% is grassland or range, and 5% is agricultural land [manual classification]. In a preliminary GIS study, all landscape elements in the research area have been identified using a manual classification of Sentinel-2 imagery. The resulting dataset contains all the research area’s water bodies, villages, roads, and other relevant elements. Most of the water area is concentrated in two large reservoirs in the northern and southern parts of the research area. The mountains form a U-shape around the study area, providing a demarcation that can potentially minimize the introduction of external materials through wind transport and other mechanisms.

### 2.2. Microplastic Sampling

The sample sites were organized into eight complexes based on location and hydrological connection (Table 1). Complex 1 included Site 1, located in a forested area with no pond connection. Complex 2 comprised Site 2.1 in an agricultural area and Site 2.2 in a horticultural area, both without pond connections, representing typical agricultural and horticultural land covers. Complex 3 included Site 3.1 in a forested area with an “Above” pond connection and Site 3.2 in a forested area with a “Below” pond connection, allowing for assessing pond connection influence in forested settings. Complex 4 consisted of Site 4 in an urban area with no pond connection, representing urban land cover. Complex 5 included Site 5 in a horticultural area without a pond connection. Complex 6 was more diverse, with Site 6.1 in a horticultural area with an “Above” pond connection, Site 6.2 with a “Below” pond connection, Site 6.3 with an “Above” pond connection, and Site 6.4 without any pond connection, allowing for a detailed comparison within horticultural settings. Complex 7 included multiple agricultural sites with varying pond connections: Site 7.1 with an “Above” pond connection, Site 7.2 with a “Below” pond connection, Site 7.3 with a “Below” pond connection, Site 7.4 with an “Above” pond connection, Site 7.5 with a “Below” pond connection, and Site 7.6 with an “Above” pond connection. This complex enabled the assessment of MP contamination in agricultural areas with different pond connections. Lastly, Complex 8 included Site 8 in an urban area without a pond connection, providing another urban comparison point.

At each of the 18 sample sites, five samples were extracted 1 m apart along the ditch bottom using new disposable wooden spoons. The sample was collected from the top centimeter of the sediment. The five samples for each site were then mixed in a glass tube, which was immediately sealed with aluminum foil.

### 2.3. MP Identification

An MP extraction methodology suitable for the research questions was applied to the ditch and pond sediments [50,51,52]. Non-plastic consumables and equipment were employed throughout the experiment. Utilized glass and metal items were washed with ultrapure water produced by Milli-Q SQ2 (Millipore, Merck KGaA, Darmstadt, Germany) and dried in an infrared fast drying box WA70-1 (Hangzhou Qiwei Instrument Co. Ltd., Hangzhou, China). Items were stored in a sealed glass box before use. Working surfaces were wiped down regularly with paper towels and ethanol alcohol, and cotton coats were worn by everyone handling the samples. Windows and doors were closed during sample handling to reduce air circulation in the laboratory.

Exactly 20 g of soil sample was combined with 60 mL of ZnCl_2_ solution (1.7–1.8 kg/L) in a 100 mL beaker, stirred for 10 min, and left to stand overnight to aid in the flotation of MP by increasing the solution’s density. Subsequently, the mixture was transferred to a new beaker, and 60 mL of 30% hydrogen peroxide (H_2_O_2_) was added to digest organic impurities. After thorough mixing, the solution was left for 24 h to allow a complete reaction of H_2_O_2_ with organic materials. The supernatant underwent vacuum filtration, and the filter membrane (0.22 μm polytetrafluoroethylene membrane) was subjected to 30 min of ultrasonication at 40 KHz in a JP-060S ultrasonic machine (Shenzhen Jiemeng Co., Ltd., Shenzhen, China) in ethanol to disperse adhered substances. After ultrasonication, the filter membrane was repeatedly rinsed with ethanol, and the solution was concentrated in the drier. The solution was then dripped on highly reflective glass slides (MY2108LD34 by Agilent Technologies Ltd., Santa Clara, CA, USA). The extracted particles were identified using laser-directed infrared spectroscopy (LDIR) utilizing an Agilent 8700 LDIR machine (Agilent Technologies Ltd., Santa Clara, CA, USA) with a 20–500 μm detection range. LDIR imaging combines infrared spectroscopy principles with high-resolution imaging capabilities, allowing for visualization of the spatial distribution of chemical compositions within the sample. This method maps heterogeneous samples with high sensitivity and spatial resolution, making it particularly useful in MP studies [53,54]. LDIR enables the counting of MP particles in a sample and the determination of the type of plastic of each particle, which is invaluable for estimating the source and transport path of the MP particles. MP particles with a match score of >0.65 were included in this study, consistent with established methodologies [55,56,57,58]. The MP counts were calculated as the MP particle count per kilogram, i.e., p/kg [59].

### 2.4. Physical and Chemical Sediment Analysis

Soil particle size distribution was determined using the Mastersizer 2000 laser particle size analyzer (Malvern Panalytical, Malvern, UK) [60]. First, to remove organic matter, approximately 0.5 g of soil sample was placed in a 150 mL glass flask and was pre-treated with 10 mL H_2_O_2_ (10%) on a heating plate under continuous washing of the flask wall to prevent foam from sticking to it. If necessary, additional H_2_O_2_ was added until no reaction was visible. This was followed by a treatment with 10 mL hydrochloric acid (HCl, 10%) at 50 °C to eliminate carbonates. Distilled water was added to reach a neutral pH, and the flask was left to stand for 24 h, after which the clear liquid was siphoned off. The remaining sample was then dispersed using 10 mL of sodium hexametaphosphate (1 mol/L) and treated for 10 min in the ultrasonic cleaning device. Then, the sample was analyzed using the laser particle size analyzer at a refractive index of 1.52. For the analysis, soil particle size classes D10, D50, and D90 were used. Each describes a diameter bigger than 10%, 50%, or 90% of the particles in the sample, respectively.

Dried soil sample was used for the determination of total organic carbon (OC). To remove inorganic carbon (IC), 10% HCl was added to the soil sample, and phosphoric acid was slowly added until there was no visible reaction. After the inorganic carbon was removed, the sample was analyzed using a Total Organic Carbon Analyzer TOC-L (Shimadzu, Kyoto, Japan). The soil’s organic matter was combusted at 900 °C inside the TOC-L, converting it to carbon dioxide (CO_2_), which was then carried by high-purity oxygen gas and detected using a non-dispersive infrared (NDIR) detector inside the TOC-L. This method ensured the accurate measurement of the total organic carbon content in the soil [61].

Organic nitrogen was calculated following the Kjeldahl methodology [62], which involves determining total nitrogen (TN) and subtracting the measured inorganic forms of nitrogen, i.e., nitrate and ammonium, from the total nitrogen content. For the determination of total nitrogen (TN), 0.5 g of air-dried soil (sieved to 0.25 mm) was placed in a Kjeldahl digestion flask (Foss Analytics, Hillerød, Denmark). After wetting the sample with a small amount of water, 4 mL of pure sulfuric acid (H_2_SO_4_) was added, and the mixture was left to soak overnight. The following day, 0.5 g of pentahydrate sodium thiosulfate was added, and the sample was heated until smoke appeared. After cooling, 1.1 g of a catalyst composed of potassium sulfate, copper sulfate pentahydrate, and titanium dioxide was added and mixed. The digestion continued until the mixture turned gray, and then it was further digested for 1 h. The digestion flask was connected to a Kjeldahl nitrogen analyzer Foss Kjeltec 8100 (Foss Analytics, Hillerød, Denmark), and the distillate was absorbed in 20 mL boric acid solution (323.47 mmol/L). After distillation, the distillate was titrated using standard hydrochloric acid solution, and the total nitrogen content was calculated based on the amount of acid consumed.

For nitrate nitrogen (NO_3_⁻-N) determination, 50 g of soil (sieved to 2 mm) was placed in a 500 mL bottle, and 0.5 g calcium sulfate and 250 mL of water were added. The bottle was then shaken for 30 min. Afterward, 25 mL of the sample solution (SS) was placed in an evaporating dish, and 0.05 g of carbonate was added. The solution was evaporated to dryness in a water bath, and after cooling, 2 mL of disulfonic acid reagent (1.18 mol/L) was added. The solution was stirred to ensure full contact with the dried material, then left to stand for 10 min. Afterward, 20 mL of water was added, and the mixture was stirred until all the dried material dissolved. While stirring, 3.71 mol/L ammonium hydroxide (1:1 ammonia solution) was slowly added until the solution became slightly alkaline (turning yellow), and an additional 2 mL of ammonia was added to ensure excess. The solution was transferred into a 100 mL volumetric flask and diluted to the mark. The nitrate nitrogen concentration was measured using a UV1800 UV-Visible Spectrophotometer (Shanghai Jinghua Science & Technology Instruments Co., Ltd., Shanghai, China) at a wavelength of 420 nm.

For ammonium nitrogen (NH_4_^+^-N) analysis, 5 mL of SS was transferred into a 50 mL volumetric flask and diluted to 10 mL with potassium chloride solution (2 mol/L). Then, 5 mL of phenol solution (10 g phenol and 100 mg sodium nitroprusside dissolved in water, diluted to 1 L) and 5 mL of alkaline sodium hypochlorite solution (10 g sodium hydroxide, 7.06 g disodium hydrogen phosphate, 31.8 g sodium phosphate dissolved in water with 10 mL of 705.25 mmol/L sodium hypochlorite solution, diluted to 1 L) was added. The mixture was shaken and left at room temperature (around 20 °C) for 1 h. After this, 1 mL of a masking agent (40 g potassium sodium tartrate and 10 g disodium EDTA dissolved in water, then mixed in equal parts) was added to dissolve any potential precipitate, and the solution was diluted to the mark with water. The ammonium nitrogen concentration was determined by measuring absorbance at a wavelength of 625 nm using the UV1800 UV-Visible Spectrophotometer.

### 2.5. Software and Data

Statistical analyses were performed in Python (version 3.9, Python Software Foundation) and the statistical programming language R (version 4.3.1, R Core Team, Vienna, Austria) in the RStudio environment (version 2024.04.1-748, RStudio, Boston, MA, USA). ArcGIS (version 10.4, Esri, Redlands, CA, USA) was utilized to create maps. The digital elevation model used for delineating the catchment area was the SRTM 90 m DEM [63,64,65,66]. Landcover classification was performed in ArcGIS using Landsat data (Landsat-9 image courtesy of the U.S. Geological Survey).

## 3. Results

### 3.1. MP Distribution in the Research Area

**Figure 2:**
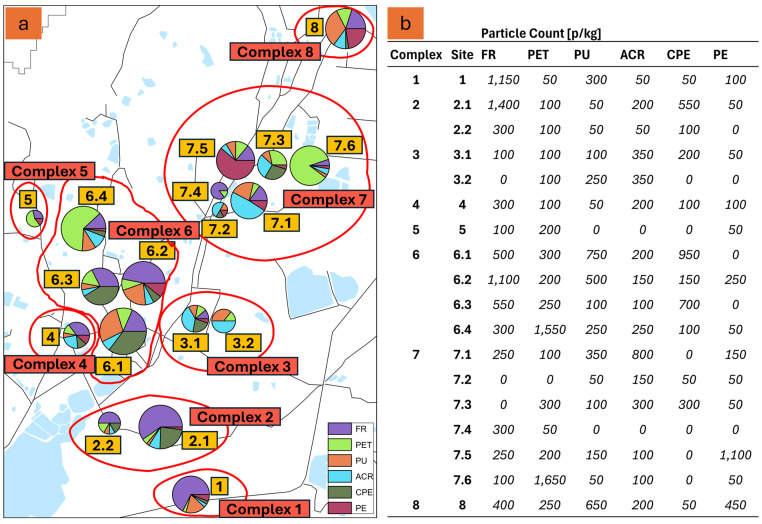
(**a**) The MP particle composition of the six most common materials for all sample sites. The diagram size indicates the overall MP count per site. (**b**) The data show the particle count for the six most common materials for all sites.

In total, 922 MP particles from 31 different materials were identified from the sediment samples (full data in Supplementary Table S1, exemplary LDIR scans in Supplementary Figures S1–S4). The six most common materials were fluororubber (FR), polyethylene terephthalate (PET), polyurethane (PU), acrylonitrile (ACR), chlorinated polyethylene (CPE), and polyethylene (PE). Figure 2 shows the distribution of these six MP over the research area (a) and the particle counts per site and material (b). The distribution of microplastics across the study area showed significant (*p* = 0.028) variations based on material type, as evidenced by the results from the different sites and complexes.

FR was the most dominant microplastic overall and in several locations, particularly at Site 1 (1150 p/kg) in Complex 1, a forested area. High FR counts were also observed at Site 2.1 (1400 p/kg) in Complex 2, an agricultural area, and Site 6.2 (1100 p/kg) in Complex 6, a horticultural area. In contrast, FR was absent in Sites 3.2, 7.2, and 7.3, indicating variability in its presence across different locations. Sites 7.1 (250 p/kg), 7.4 (300 p/kg), and 7.5 (250 p/kg) in Complex 7 showed moderate levels of FR, while Site 8, an urban area in Complex 8, contained 400 p/kg of FR. PET showed the highest counts in Site 6.4 (1550 p/kg) within Complex 6, a horticultural area, and Site 7.6 (1650 p/kg) in Complex 7, an agricultural site. PET was also detected in smaller quantities across multiple locations, including Sites 2.1 and 2.2 in Complex 2, Sites 3.1 and 3.2 in Complex 3, and Site 4 in Complex 4, each with 100 p/kg. PU was found in notable amounts at Site 6.1 in Complex 6, with 750 p/kg, and Site 8 in Complex 8, with 650 p/kg. PU was also present at Site 7.1 in Complex 7 (350 p/kg), while smaller quantities were detected at other locations, such as Sites 3.1 and 3.2 in Complex 3 and Sites 2.1 and 2.2 in Complex 2. PU was absent at Sites 7.4 and 7.5 in Complex 7. ACR was most prevalent at Site 7.1 in Complex 7, where 800 p/kg were found. It was also present at Sites 3.1 and 3.2 in Complex 3, with 350 p/kg each, and at Site 4 in Complex 4 (200 p/kg). Other sites, including Sites 2.1, 6.1, and 8, showed smaller amounts of ACR. CPE exhibited notable concentrations at Site 6.1 in Complex 6, with 950 p/kg, and at Site 6.3, also in Complex 6, with 700 p/kg. Other sites, such as 2.1 in Complex 2 and 3.1 in Complex 3, had smaller amounts of CPE (550 p/kg and 200 p/kg, respectively). CPE was absent at several sites, including Sites 7.4 and 7.5 in Complex 7. PE was most abundant at Site 7.5 in Complex 7, where 1100 p/kg were recorded. PE was also present at Site 8 in Complex 8 (450 p/kg) and Site 7.1 (150 p/kg). In contrast, many sites, including 6.1 in Complex 6 and 7.4 in Complex 7, had no detectable PE particles.

### 3.2. Microplastic Particle Count Clustering

A k-means clustering analysis was performed to identify similar distribution patterns of different MP types. Acceptable clustering performances (measured by a silhouette score of >0.6) were obtained from clustering FR and PET particle counts and CPE and PU particle counts. Table 2 shows the clustering results for all 18 sampling sites.

In the FR/PET clustering, most sites were grouped into either Cluster FR/PET_H/L or Cluster FR/PET_L/L, with fewer sites falling into Cluster FR/PET_L/H. Cluster FR/PET_H/L, characterized by high FR and low PET counts, included Site 1 and Site 2.1. At Site 1, located in a forested area, FR dominated with 1150 p/kg, while PET was minimal (50 p/kg). Similarly, Site 2.1, in an agricultural area, had 1400 p/kg and only 100 p/kg PET particles. Cluster FR/PET_L/L represented sites such as 2.2, 3.1, 3.2, and 4, where both FR and PET were found in moderate to low concentrations. For instance, Site 2.2 had 300 p/kg FR particles and 100 p/kg PET, while Site 3.1 and Site 3.2, located in forested areas with pond connections, each had 100 p/kg PET but a smaller number of FR particles (100 p/kg and 0 p/kg, respectively). Site 4, representing an urban area, also showed low levels of both FR (300 p/kg) and PET (100 p/kg). Cluster FR/PET_L/H, comprising Sites 6.4 and 7.6, exhibited the opposite pattern, with high-PET counts and low FR counts. Site 6.4, a horticultural area, contained 1550 p/kg PET and 300 p/kg FR, while Site 7.6, an agricultural site, showed 1650 p/kg PET and only 100 p/kg FR.

In the CPE/PU clustering, the sites were more evenly distributed between Cluster CPE/PU_H/H and Cluster CPE/PU_L/L. Cluster CPE/PU_H/H, with higher concentrations of CPE and PU, included Sites 2.1, 6.1, and 6.3. For instance, Site 2.1, located in an agricultural area, had 550 p/kg CPE and 50 p/kg PU. Similarly, horticultural sites such as 6.1 and 6.3 displayed elevated levels of CPE and PU, with Site 6.1 having 950 p/kg CPE and 750 p/kg PU. Cluster CPE/PU_L/L encompassed most other sites, including Sites 1, 2.2, 3.1, 3.2, and 4, which all had lower levels of CPE and PU. For example, Site 1 had 50 p/kg CPE and 300 p/kg PU, Site 2.2 had 100 p/kg CPE and 50 p/kg PU, and Sites 3.1 and 3.2 contained low quantities of both CPE and PU.

### 3.3. Interactions of MPs with Ponds

The distribution of MP in drainage ditches showed significant differences (*p* < 0.001) depending on their connection to ponds, with differences observed between sites sampled directly above ponds, below ponds, and those without significant pond connections (Table 3). These variations offer insights into how ponds influence MP retention and transport.

Sites with “Above” pond connections generally exhibited higher MP concentrations than those with “Below” pond connections or sites without pond influence. For example, Site 6.1 (horticulture, above a pond) displayed elevated levels of FR and CPE, with 500 p/kg and 950 p/kg particles, respectively. Similarly, Site 7.6 (agriculture, above a pond) exhibited a large amount of PET, with 1650 p/kg. The average MP count in these sites was 1700 p/kg, indicating that ditches above ponds accumulated higher concentrations of MP. In contrast, sites with “Below” pond connections, such as Sites 3.2, 6.2, and 7.2, showed lower MP concentrations, particularly for FR. For instance, Site 3.2 (forest, below a pond) had no detectable FR particles, and Site 7.2 (agriculture, below a pond) also showed an absence of FR particles. The average MP count in these sites was lower at 1050 p/kg. Sites without significant pond connections showed variable MP concentrations. For instance, Site 2.1 (agriculture, no pond connection) exhibited the highest concentration of FR (1400 p/kg), while Site 6.4 (horticulture, no pond connection) had a notably high concentration of PET (1550 p/kg). The average MP count in sites without pond connections was 1250 p/kg.

### 3.4. MP Relationship with Land Cover

The analysis showed a significant (*p* < 0.001) difference in the distribution of MP types across different land cover types, indicating that land use considerably influences MP distribution. The study examined four land cover types, consisting of agriculture, horticulture, forest, and urban. The data revealed distinct patterns in the presence of various MP types, including FR, PET, PU, ACR, CPE, and PE. Agricultural sites (e.g., 2.1, 7.1–7.6) exhibited notably higher concentrations of FR and PET, with FR counts reaching up to 1400 p/kg at Site 2.1 and up to 1650 p/kg at Site 7.6. Horticultural areas (Sites 6.1–6.4) also showed significant PET, PU, and CPE concentrations. In contrast, forested sites (1, 3.1, and 3.2) generally had lower MP counts, although Site 1 had a relatively high concentration of FR particles (1150 p/kg). Urban sites (4 and 8) exhibited a more diverse MP profile, with notable counts of PU and PE.

### 3.5. MP Shape Parameter Relationship with Soil Properties

The analysis of the relationship between MP shape parameters and soil properties revealed several statistically significant correlations (*p* < 0.05), highlighting potential associations between specific MP characteristics and soil features. Table 4 presents the ten strongest correlations observed between MP shape parameters—such as solidity (a measure of how filled in or compact a particle is), eccentricity (a measure of the elongation of the particle), circularity (a measure of how circular the particle is), diameter, area, width, longest side, and height—and soil properties, including D90 (the particle size at which 90% of the soil sample is finer), D50 (the median particle size), organic carbon (OC), and organic nitrogen (ON). A full table of correlations is available in Supplementary Table S2.

Among the strongest correlations, ACR showed a positive relationship between solidity and the D90 soil particle size parameter (coefficient = 0.58). This suggests that MPs with more compact and filled shapes are more commonly associated with soils containing larger particles. Similarly, ACR eccentricity and circularity were positively correlated with D50 (r = 0.45) and D90 (r = 0.42), respectively, indicating that elongated and more circular MPs are associated with specific soil texture characteristics. For PE, several shape parameters, such as diameter, area, and width, were negatively correlated with organic carbon (r = −0.39 for diameter and area) and organic nitrogen (r = −0.39 for diameter and area; r = −0.38 for width and longest side), implying that larger PE particles tend to occur in soils with lower organic content. Finally, a positive correlation (r = 0.37) was observed between PU height and D90, suggesting a relationship between PU particle height and larger soil particles.

## 4. Discussion

### 4.1. Sources of MP Particles

The distribution of MPs across the study area revealed distinct variations that correspond to different land use practices and local sources of pollution. These variations highlight how the environmental context—such as urban, agricultural, or forested areas—affects the prevalence of certain MP types with clear ties to human activities.

Rubber-based MPs like FR were predominantly found in areas affected by road traffic. This is consistent with the observation that FR is commonly released through tire wear during driving, which introduces MPs into the environment via road runoff and airborne dust [67]. In forested areas near roads, this explains the high levels of FR, which may be further exacerbated by industrial activities involving seals and gaskets [68]. These findings suggest that proximity to roads and industrial zones significantly determines FR prevalence, particularly in Complex 1. However, Complexes 4–7, although also bordered by roads, show significantly lower levels of fluororubber. This discrepancy could be influenced by several factors, including the distance from the road, the presence of barriers such as drainage ditches, or local environmental conditions like wind patterns and rainfall. These variations underscore the complexity of MP transport mechanisms and highlight the need for further investigation into how such factors influence MP deposition. Polymer-based MPs, including PET, PE, and CPE, show a strong association with both urban and agricultural land use. PET particles are often linked to synthetic textiles, such as polyester, which release fibers into wastewater during washing [69]. This pattern is particularly evident in agricultural areas where synthetic textiles, plastic packaging, and agricultural films contribute to PET contamination [70]. In Complexes 6 and 7, the abundance of PET suggests significant agricultural use of plastic-based products, such as mulching films and packaging, as well as wastewater pathways that introduce these MPs into the environment [71]. Similarly, PE MPs, prevalent in urban environments, likely originate from consumer products such as cosmetics [72] and the degradation of larger plastic items like bags and packaging. PE in urban areas like Complex 4 underscores the importance of plastic waste management in minimizing MP pollution. PE in cosmetic products and packaging explains its widespread presence in urban and agricultural settings, reflecting how everyday consumer products contribute to environmental contamination. PU plays a different role in agricultural settings, particularly due to its use in pesticide microcapsules and coatings [73]. The high levels of PU observed in Complexes 6 and 7 suggest that agricultural practices involving pesticides and synthetic coatings are a significant source of this MP. Additionally, PU is released by degrading materials such as synthetic leathers and road markings, which can further contribute to pollution in nearby environments [74]. CPE, often used in industrial applications like roofing membranes and hoses, is also associated with agricultural and urban areas. The presence of CPE in Complexes 2 and 6 highlights the impact of agricultural plastic materials and industrial runoff, where these products degrade and release MPs into the surrounding environment [75]. Similar to PE, the degradation of CPE through exposure to UV radiation and environmental factors contributes to its persistence in terrestrial and aquatic environments [76]. Lastly, ACR MPs are commonly linked to textile fibers, especially in areas with high human activity. The detection of ACR in both rural and urban areas suggests that these MPs are widespread due to atmospheric deposition and runoff from synthetic clothing and coatings [77].

These findings show that MP pollution is highly context-dependent, with the type and concentration of MPs varying significantly based on local sources. Furthermore, the relatively high concentration of microplastics (MPs) in forest ditches points to the role of these ditches as accumulation zones. While site selection aimed to minimize the influence of adjacent areas, temporary hydrological connections between the forested ditches and neighboring regions (e.g., agricultural fields, urban areas, or roads) may lead to runoff carrying MPs into these supposedly isolated areas. This suggests that even forested areas can be affected by external sources of pollution, especially during certain environmental conditions such as heavy rain. The clear connection between road traffic and FR concentrations indicates the need for targeted mitigation efforts in locations near infrastructure. At the same time, the prevalence of PET and PE in both agricultural and urban areas underscores the impact of plastic use in everyday products and agricultural practices. Agricultural sites are particularly affected by PU and CPE, likely due to their role in pesticides and industrial products. This raises concerns about the long-term environmental impacts of these materials, especially as they persist in the soil and can interact with other pollutants [73]. In urban areas, the diversity of MP sources—including consumer products, textiles, and construction materials—highlights the complexity of addressing MP pollution. These findings emphasize the importance of managing plastic use in urban and rural settings. By understanding MPs’ specific sources and pathways, targeted strategies can be developed to reduce their environmental impact, particularly in areas most affected by industrial, agricultural, or urban activities.

### 4.2. MP Source Patterns

The clustering results highlighted distinct patterns in the distribution of MPs across different land use types, demonstrating how source activities and transport processes interact to shape MP presence in the environment. These patterns, characterized by specific combinations of MP types such as FR, PET, CPE, and PU, provide insights into the complex dynamics between sources, environmental conditions, and MP transport.

In Cluster FR/PET_H/L (high FR, low PET), the high FR concentrations reflect road traffic’s significant role in introducing FR MPs into the environment [67]. The low PET concentrations in these areas suggest that while road traffic is a dominant source of FR, it does not contribute significantly to PET pollution. The transport of FR via runoff and its retention in nearby soil or sediment further emphasize the role of roads as pathways for this type of MP, particularly in forested areas like Complex 1. The relative isolation of these environments from urban centers may also limit the introduction of PET from urban-related sources like synthetic textiles and packaging. Conversely, Cluster FR/PET_L/H (low FR, high PET) highlights the dominance of PET in agricultural and horticultural environments, where synthetic textiles, plastic packaging, and agricultural films are common [69,70]. The high PET concentrations in these areas, particularly in Complexes 6 and 7, point to the influence of both local sources—such as agricultural plastics—and transport mechanisms, including surface runoff from agricultural fields and wastewater pathways. The interaction between PET sources and these transport routes creates an environment where PET is more readily retained and accumulates over time. FR remains limited due to the absence of substantial road traffic or industrial activity.

The CPE/PU clusters reveal further complexities in MP source and transport interactions. Cluster CPE/PU_H/H (high CPE, high PU) predominantly features agricultural and horticultural sites, such as Sites 2.1, 6.1, and 6.3 high in CPE and PU. These materials are commonly used in products such as plastic membranes, hoses, and pesticide microcapsules [73,75], reflecting these sites’ management and use practices. These MPs likely enter the environment through direct degradation and runoff, but local environmental factors like sediment characteristics and water flow influence their persistence in the soil. Agricultural practices that disturb the soil and alter water retention could enhance CPE and PU deposition and retention, contributing to higher concentrations in these areas. CPE and PU in high quantities indicate how land use practices can co-locate different MP types through shared transport mechanisms, such as irrigation runoff and agricultural drainage systems. In contrast, Cluster CPE/PU_L/L (low CPE, low PU) represents areas with lower agricultural and industrial activity, including forested and urban environments where these materials are minimal, such as Site 1 and Site 4. These areas lack the direct sources of CPE and PU, and the transport processes present—such as natural surface runoff in forests—are less likely to carry and deposit these MPs. The absence of agricultural practices reduces the pathways through which these MPs could be introduced and retained, resulting in lower concentrations.

The clustering results emphasize how different MP source and transport processes interact to create the specific source patterns observed in different environmental contexts. These patterns are shaped not only by the presence of specific sources but also by the environmental conditions and transport mechanisms that determine where and how MPs are retained. These findings align with previous research showing that MP distribution is highly influenced by both land use and the interaction of transport processes with MP sources [22,23,24,25,37].

### 4.3. MP Interactions with Ponds

The results revealed that pond connections significantly influence the distribution and retention of microplastics in drainage ditches. In sites with “Above” pond connections, higher concentrations of MP, particularly FR and PET, were observed. These elevated MP counts suggest that ditches above ponds accumulate MPs from upstream sources, such as road runoff or agricultural inputs, and transport them toward ponds. The higher average MP count (1700 p/kg) in these areas indicates that ponds serve as critical points where MPs are likely to settle or be retained before entering downstream aquatic systems. In contrast, sites with “Below” pond connections exhibited lower MP concentrations, especially for heavier materials like FR. The absence of FR particles at Sites 3.2 and 7.2 suggests that ponds act as sinks, effectively capturing heavier microplastics such as FR. This reduction in MPs downstream of ponds aligns with findings from other studies, which have demonstrated the role of ponds in filtering and retaining specific pollutants and materials in agricultural landscapes [78,79]. The lower average MP count (1050 p/kg) in sites below ponds supports the hypothesis that ponds play a significant role in MP retention. It is noteworthy here that many MP particles have, depending on the material, a lower density than water [80], causing them to be buoyant in water. Therefore, two processes of MP particle retention in ponds are feasible. First, the weight of MP particles may be increased through biofilm formation and aggregation with inorganic and organic matter [81,82]. This process may already begin while larger plastic objects are breaking down in the soil, contributing to the eventual formation of MPs. A second process of MP particle retention in ponds may be the ingestion by fish and aquatic invertebrates [3,4,5] and adsorption and accumulation in aquatic vegetation [83,84]. For sites without pond connections, the results were more variable. While some sites, like 2.1, had high concentrations of FR, others, such as 6.4, showed elevated PET levels. This variability suggests that microplastics are less likely to be retained in the absence of ponds and may accumulate in ditches or be transported further downstream.

This study reinforces the broader role of ponds in agricultural ecosystems, not merely as passive water bodies but as active agents in reducing MP pollution. Ponds below ditches, in particular, demonstrate their capacity to trap heavier MPs, thereby limiting the dispersal of pollutants downstream. The contrast between “Above” and “Below” pond connections reveals a clear dynamic, where ditches funnel MPs toward ponds, and the ponds, in turn, significantly reduce the downstream movement of certain materials. This function of ponds as retention zones is vital for developing strategies to mitigate the environmental impacts of microplastic pollution in agricultural landscapes, echoing insights from related studies on pollutant management [85,86,87,88].

### 4.4. MP Interactions with Land Cover

Land use not only acts as a source of MP pollution but also influences the pathways through which these particles are transported and accumulated. Different land use types introduce various patterns of runoff, management practices, and human activities, all of which affect how MPs enter and persist in the environment [89,90]. For instance, agricultural and urban areas at Sites 4 and 7.1 may generate high volumes of runoff containing MPs from fertilizers, plastics, and road materials. In contrast, forested areas, such as Complexes 1 and 3, may experience less direct contamination but still collect MPs from surrounding infrastructure. These patterns are shaped by land use decisions, including water management, soil conservation practices, and the intensity of human activity [91,92]. In this context, land use becomes a multifaceted factor that contributes to the generation of MP pollution and dictates how these pollutants move through and impact different ecosystems. The significant variation in MP types across different land cover types observed in this study aligns with findings from previous research [93,94], highlighting the role of human activity not only in the initial sources of MP pollution but also in the fate of MP particles. These results indicate the need for more sustainable agricultural practices that reduce reliance on plastic-based materials. The horticultural sites 6.3 and 6.4, where PET, PU, and CPE were more prominent, illustrate another case where plastic use, particularly in irrigation systems, coverings, and pesticide containers, contributes to environmental contamination. The frequent use of plastic-based products in these settings likely accounts for the higher concentrations of these MPs, which could persist in the environment long after use. The forest Sites 3.1, 3.2, and 1 typically had lower MP concentrations, but the high FR levels at Site 1 suggest localized pollution, possibly from nearby road traffic. This highlights how even remote or natural areas are not immune to MP contamination, especially when close to infrastructure. Urban areas, with their varied MP profiles, reflect the complexity of sources contributing to pollution. The elevated levels of PU and PE at Site 8 suggest a mixture of inputs. This diversity of MP types in urban environments underscores the need for better waste management strategies and infrastructure to mitigate plastic pollution. The findings suggest that land use is critical in determining the type and concentration of MPs in the environment. These insights can inform targeted mitigation strategies based on specific land use practices and associated risks to reduce MP pollution.

### 4.5. MP Shape Parameter Interactions with Soil Properties

The correlations observed between MP shape parameters and soil properties provided valuable insights into potential relationships, although these findings do not imply causality. The strong positive correlation between ACR solidity and soil particle size (D90) suggests that more compact and filled ACR particles may preferentially accumulate in coarser soils. This relationship could indicate that the soil’s physical structure affects the retention or movement of MP particles with simpler, more solid shapes. Similarly, the positive relationships between ACR eccentricity and circularity with soil particle size parameters (D50 and D90) suggest that elongated or circular MPs may persist in soils with specific textures, perhaps due to the interactions between soil particles and the shape of the MPs. Elsewhere, the reduced mobility and increased retention of larger MP particles in soils have been demonstrated [95,96]. The negative correlations between PE particle size parameters (e.g., diameter, area, width) and organic content (both organic carbon and nitrogen) suggest that larger PE particles are less likely to be found in soils with higher organic content. This may be due to the ability of organic matter to better retain smaller particles. Other studies have found a strong effect of OC on MP particle sorption [97]. Alternatively, organic matter may be associated with different sources or behaviors of MPs in the soil, leading to a lower prevalence of larger MP particles in organic-rich environments. Microplastic has even been demonstrated to bond with OC aggregates [98]. The positive correlation between PU height and D90 indicates that longer PU particles are more likely to be associated with larger soil particles. This could suggest that PU particles behave differently in coarser soils, potentially influenced by the soil structure, water movement, or other factors affecting MP retention and movement. However, since these correlations point to relationships rather than causal mechanisms, further research is needed to explore the underlying processes driving these associations. Understanding how soil properties interact with MP characteristics is critical for predicting MP behavior in different environments and could inform more effective management practices for mitigating MP contamination.

### 4.6. Limitations

This study offers insights into microplastic (MP) distribution and sources in a small agricultural catchment in East China, but some limitations should be noted. The limited scope of sample sites may affect the generalizability of our results to regions with different environmental conditions or land use practices. Furthermore, the sampling methodology focused only on the top 1 cm of sediment, which may not capture the full vertical distribution of MPs, especially in areas with high sedimentation rates. Additionally, temporal variability was not considered, as samples were collected at a single time point. Seasonal changes and varying agricultural practices could influence MP concentrations over time. Lastly, MP particles in environmental samples undergo aging processes, often contain chemical additives, and need chemical treatment to be isolated. Therefore, similar studies employing the same equipment for soil and sediment studies include particles with an LDIR matching score of >0.65. This means there may be some uncertainty in the chemical identification of particles within this range, potentially leading to misclassification.

## 5. Conclusions

This study provides critical insights into the distribution, sources, and transport dynamics of MP in drainage ditches in an East China catchment. The findings show significant variation in MP concentrations across different land uses, with agricultural and horticultural areas contributing the highest levels of PET, PU, and CPE. In contrast, forests and urban areas displayed a more diverse range of MP sources in this area. The influence of pond connectivity on MP transport was evident from the analyses, and ponds seem to act as sinks for certain MP. The specific processes associated with the accumulation of MP particles in ponds, such as biofilm formation and adsorption by aquatic plants, require further study. The analysis highlighted patterns, linking MP types to local sources such as road traffic, agricultural machinery, and plastic use in farming practices. The correlations between soil properties and MP shape parameters further suggest that soil composition plays a role in the retention and mobility of MP in agricultural landscapes. The findings of this study highlight the need for targeted management strategies to reduce MP pollution in agricultural landscapes. One key implication for the research area is the importance of improving agricultural practices, such as reducing the use of plastic materials in farming (e.g., mulching films and plastic packaging) and promoting biodegradable alternatives. Furthermore, the results showed that MP pollution is highly site-specific and dependent on multiple factors, which still need intense study. Additionally, regular maintenance of drainage ditches and rural ponds, including sediment removal, can help mitigate the accumulation of MP. Road runoff management, particularly near agricultural areas, should also be prioritized to minimize MP input from tire wear and industrial materials.

## Figures and Tables

**Table 1:** The complexes with their associated sample sites, as well as the land cover type and pond connection of each sampling site.

Complex	Site	Land Cover	Pond Connection
1	1	Forest	None
2	2.1	Agriculture	None
	2.2	Horticulture	None
3	3.1	Forest	Above
	3.2	Forest	Below
4	4	Urban	None
5	5	Horticulture	None
6	6.1	Horticulture	Above
	6.2	Horticulture	Below
	6.3	Horticulture	Above
	6.4	Horticulture	None
7	7.1	Agriculture	Above
	7.2	Agriculture	Below
	7.3	Agriculture	Below
	7.4	Agriculture	Above
	7.5	Agriculture	Below
	7.6	Agriculture	Above
8	8	Urban	None

**Table 2:** Clustering of FR/PET and CPE/PU particle counts for all sample sites.

Complex	1	2		3		4	5	6				7						8
Site	1	2.1	2.2	3.1	3.2	4	5	6.1	6.2	6.3	6.4	7.1	7.2	7.3	7.4	7.5	7.6	8
FR/PET	H/L	H/L	L/L	L/L	L/L	L/L	L/L	L/L	H/L	L/L	L/H	L/L	L/L	L/L	L/L	L/L	L/H	L/L
CPE/PU	L/L	H/H	L/L	L/L	L/L	L/L	L/L	H/H	L/L	H/H	L/L	L/L	L/L	L/L	L/L	L/L	L/L	L/L

Note: H = high, L = low, combination indicates which material is abundant at the respective site.

**Table 3:** Pond connection information for all sample sites.

Pond Connection	Sites	Total MP Particle Count	Average MP Count per Site [p/kg]
Above	3.1, 6.1, 6.3, 7.1, 7.4, 7.6	204	1700
Below	3.2, 6.2, 7.2, 7.3, 7.5	105	1050
None	1, 2.1, 2.2, 4, 5, 6.4, 8	176	1250

**Table 4:** The ten strongest, statistically significant correlations observed between MP particle shape parameters and soil parameters.

Material	Shape Parameter	Soil Parameter	Coefficient
ACR	Solidity	D90	0.58
	Eccentricity	D50	0.45
	Circularity	D90	0.42
PE	Diameter	OC	−0.39
	Area	OC	−0.39
	Diameter	ON	−0.39
	Area	ON	−0.39
	Width	ON	−0.38
	Longest Side	ON	−0.38
PU	Height	D90	0.37

## Data Availability

The original contributions presented in the study are included in the article or Supplementary Material. Further inquiries can be directed to the corresponding author.

## Supplementary Materials

The following supporting information can be downloaded at: https://www.mdpi.com/article/10.3390/toxics12100761/s1, Supplementary Table S1: Full data set of soil properties and microplastic particle counts in particles per kilogram [p/kg]; Supplementary Table S2: The statistically significant correlations observed between MP particle shape parameters and soil parameters. Supplementary Figure S1: The LDIR identification image of the sample from Site 6.3; Supplementary Figure S2: The LDIR identification image of the sample from Site 6.4; Supplementary Figure S3: A fluororubber (FR) particle identified in the sample from Site 7.1; Supplementary Figure S4: A polyethylene (PE) particle identified in the sample from Site 6.2.
