# Supplementary material for: Microplastic Transport and Accumulation in Rural Waterbodies: Insights from a Small Catchment in East China
Source: Toxics. 2024 Oct 19;12(10):761. doi: 10.3390/toxics12100761 (PMC11510974; doi:10.3390/toxics12100761)

Supplementary Table S1. Full data set of soil properties and microplastic particle counts in particles per kilogram [p/kg].

Site	Complex	OC	ON	D10	D50	D90	FR	PET	PU	ACR	CPE	PE	PP	PTFE	PVC	PMMA	CPI	PB
1	1	12.1	1.958	7.01	224	339.5	1,150	50	300	50	50	100	0	400	0	0	750	0
2.1	2	8.1	0.884	3.95	19.6	239.9	1,400	100	50	200	550	50	0	200	200	100	100	1,500
2.2	2	18.3	2.313	2.805	11.35	278.8	300	100	50	50	100	0	0	0	0	150	0	0
3.1	3	62.7	2.318	4.77	13.67	305	100	100	100	350	200	50	0	50	50	100	0	50
3.2	3	83.2	2.247	3.66	11	48.49	0	100	250	350	0	0	50	0	0	250	0	0
4	4	26.9	5.693	9.9	230.6	309.9	300	100	50	200	100	100	100	0	100	100	0	0
5	5	15.4	2.713	3.29	41.3	231.1	100	200	0	0	0	50	0	200	50	50	50	0
6.1	6	11.4	1.663	3.52	11.22	39.86	500	300	750	200	950	0	50	0	300	50	100	0
6.2	6	38	2.997	2.431	7.46	27.88	1,100	200	500	150	150	250	1,050	0	150	100	150	0
6.3	6	50	4.106	3.28	13.32	250.5	550	250	100	100	700	0	50	150	250	200	50	0
6.4	6	17.7	6.361	2.807	11.96	43	300	1,550	250	250	100	50	0	0	200	100	100	0
7.1	7	15.3	1.83	2.969	15.57	308.7	250	100	350	800	0	150	50	100	0	200	0	0
7.2	7	23.3	2.755	4.17	11.22	36.21	0	0	50	150	50	50	0	0	150	0	0	0
7.3	7	28.5	2.441	4.34	20.93	270.2	0	300	100	300	300	50	0	0	150	50	0	0
7.4	7	25.1	1.859	7.47	185	298.5	300	50	0	0	0	0	0	350	0	150	0	0
7.5	7	16.5	2.082	5.04	40.14	309.9	250	200	150	100	0	1,100	250	250	50	0	150	50
7.6	7	13.4	1.615	3.27	12.175	36.14	100	1,650	50	100	0	50	550	0	50	50	50	0
8	8	9.5	1.11	3.935	20.305	229.9	400	250	650	200	50	450	0	200	150	200	300	0

Supplementary Table S1 (continuation). Full data set of soil properties and microplastic particle counts in particles per kilogram [p/kg].

Site	Complex	PC	FSR	PLA	EAA	PIB	PR	PS	BR	PVB	PLS	PEP	POM	SBR	MBS	EVA	SBS	SIS	PBAT	PIM
1	1	950	0	50	0	0	150	0	0	50	50	50	50	50	0	50	0	0	0	0
2.1	2	0	300	150	50	0	0	0	0	0	0	0	0	0	0	0	50	50	0	0
2.2	2	50	0	0	0	0	50	0	0	0	0	0	0	0	50	0	0	0	0	0
3.1	3	0	0	0	150	0	0	0	100	50	0	0	0	0	0	0	0	0	0	50
3.2	3	0	0	0	50	0	0	50	0	0	0	0	0	0	0	0	0	0	0	0
4	4	50	50	0	0	0	0	50	50	200	0	50	50	0	0	0	0	0	0	0
5	5	0	0	50	50	0	100	0	0	0	0	0	0	0	0	0	0	0	150	0
6.1	6	0	200	0	50	0	0	0	0	0	50	0	50	50	0	100	0	0	0	0
6.2	6	0	50	100	50	700	0	0	50	0	0	0	50	0	50	0	0	0	0	0
6.3	6	0	150	150	100	0	0	0	50	0	0	0	50	50	0	0	50	0	0	0
6.4	6	0	0	0	100	0	0	50	0	0	0	150	0	0	0	0	0	0	0	0
7.1	7	0	0	0	0	0	0	0	0	0	0	50	0	0	50	0	0	0	0	0
7.2	7	0	0	0	0	0	0	0	0	50	0	0	0	0	0	0	0	0	0	0
7.3	7	0	0	0	100	0	0	0	50	0	0	0	0	0	0	0	0	50	0	0
7.4	7	150	50	50	0	0	0	100	0	50	0	0	0	0	50	0	0	0	0	0
7.5	7	100	100	150	0	0	100	300	0	50	200	100	0	0	0	0	0	50	0	0
7.6	7	0	0	0	0	0	0	0	0	0	0	0	0	0	0	0	0	0	0	0
8	8	0	150	150	100	0	250	50	250	0	100	0	50	100	0	0	50	0	0	0

Supplementary Table S2. The statistically significant correlations observed between MP particle shape parameters and soil parameters.

Material	Shape Parameter	Soil Parameter	Coefficient
FR	Solidity	D90	0.26
PU	Height	D50	0.27
	Height	D90	0.37
	Longest Side	D90	0.26
	Shortest Side	D90	0.27
	Diameter	D90	0.29
	Eccentricity	D50	0.3
	Eccentricity	D90	0.3
CPE	Width	OC	0.29
	Width	ON	0.29
	Width	D50	-0.33
	Height	OC	0.27
	Shortest Side	OC	0.34
	Shortest Side	ON	0.33
	Perimeter	OC	0.3
	Perimeter	ON	0.29
ACR	Eccentricity	D50	0.45
	Circularity	D90	0.42
	Solidity	D90	0.58
PE	Width	OC	-0.37
	Height	OC	-0.32
	Longest Side	OC	-0.35
	Shortest Side	OC	-0.34
	Diameter	OC	-0.39
	Area	OC	-0.39
	Perimeter	OC	-0.36
	Width	ON	-0.38
	Height	ON	-0.32
	Longest Side	ON	-0.38
	Shortest Side	ON	-0.33
	Diameter	ON	-0.39
	Area	ON	-0.39
	Perimeter	ON	-0.37

Supplementary Figure S1: The LDIR identification image of the sample from Site 6.3.

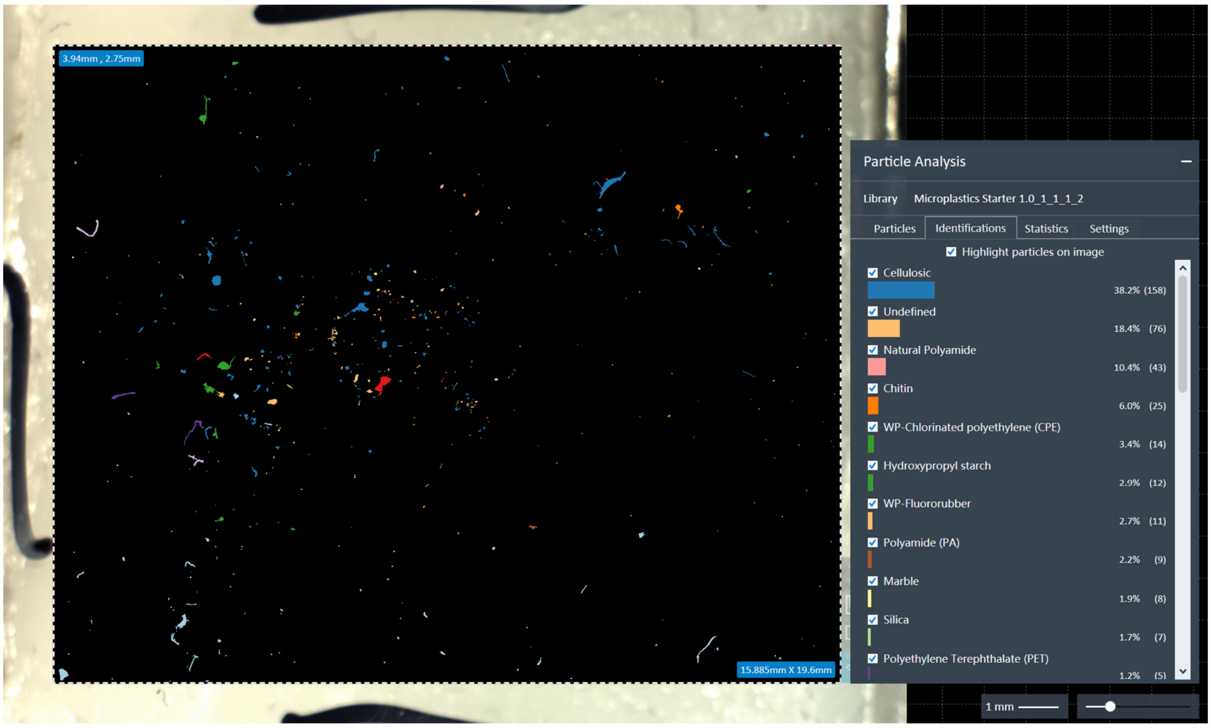

Supplementary Figure S2: The LDIR identification image of the sample from Site 6.4.

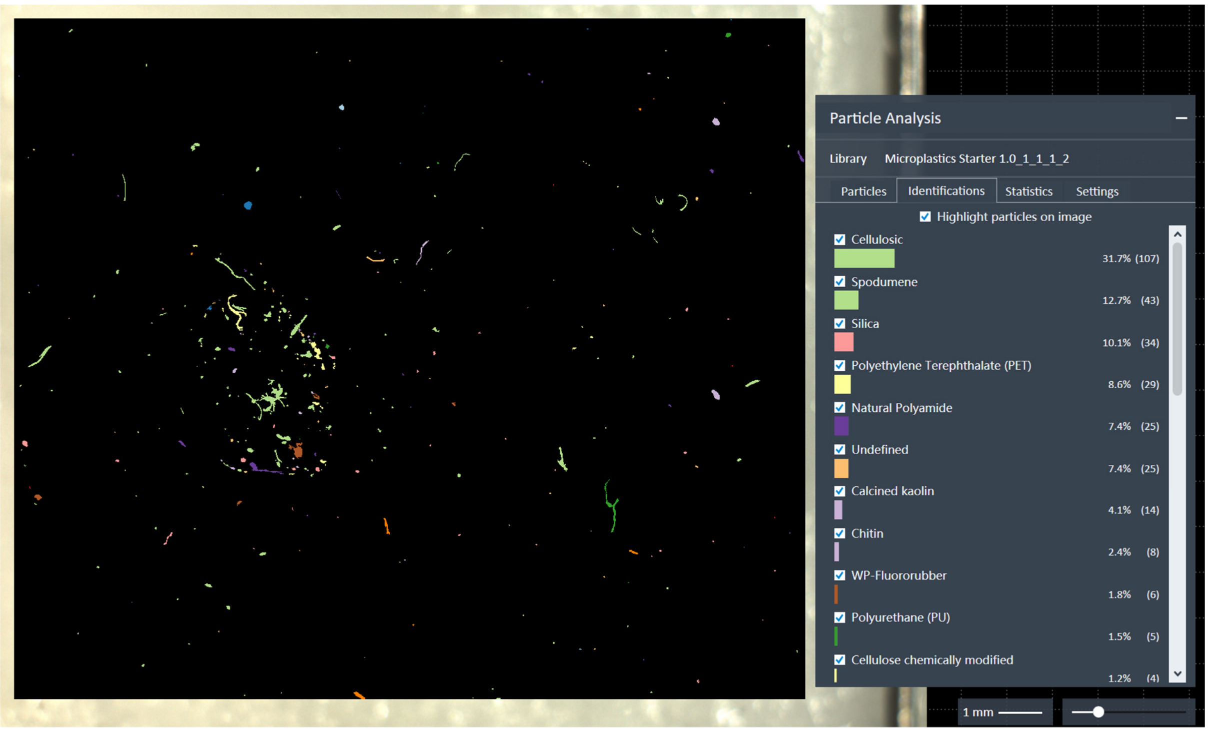

Supplementary Figure S3: A Fluororubber (FR) particle identified in the sample from site 7.1.

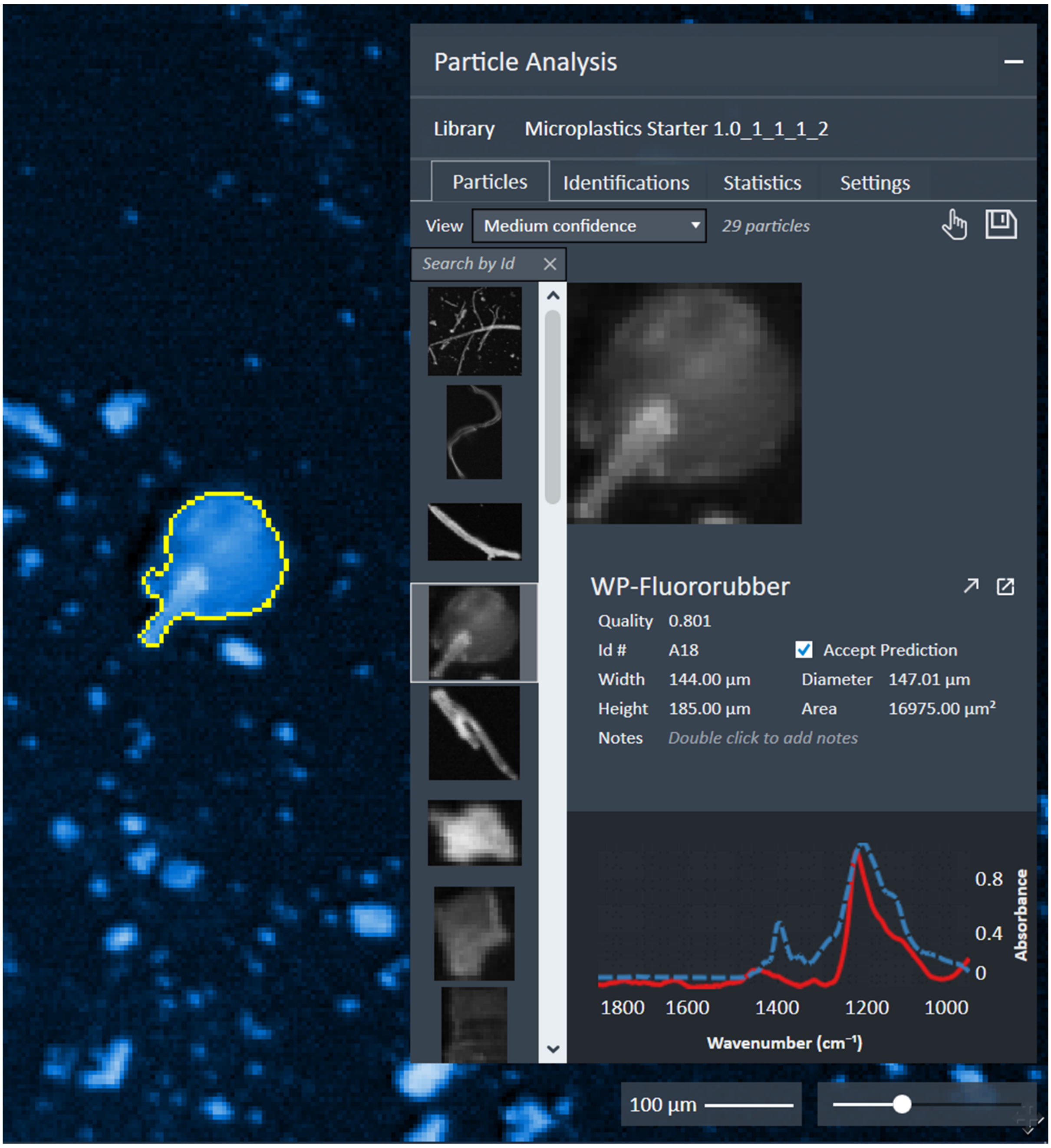

Supplementary Figure S4: A Polyethylene (PE) particle identified in the sample from site 6.2.

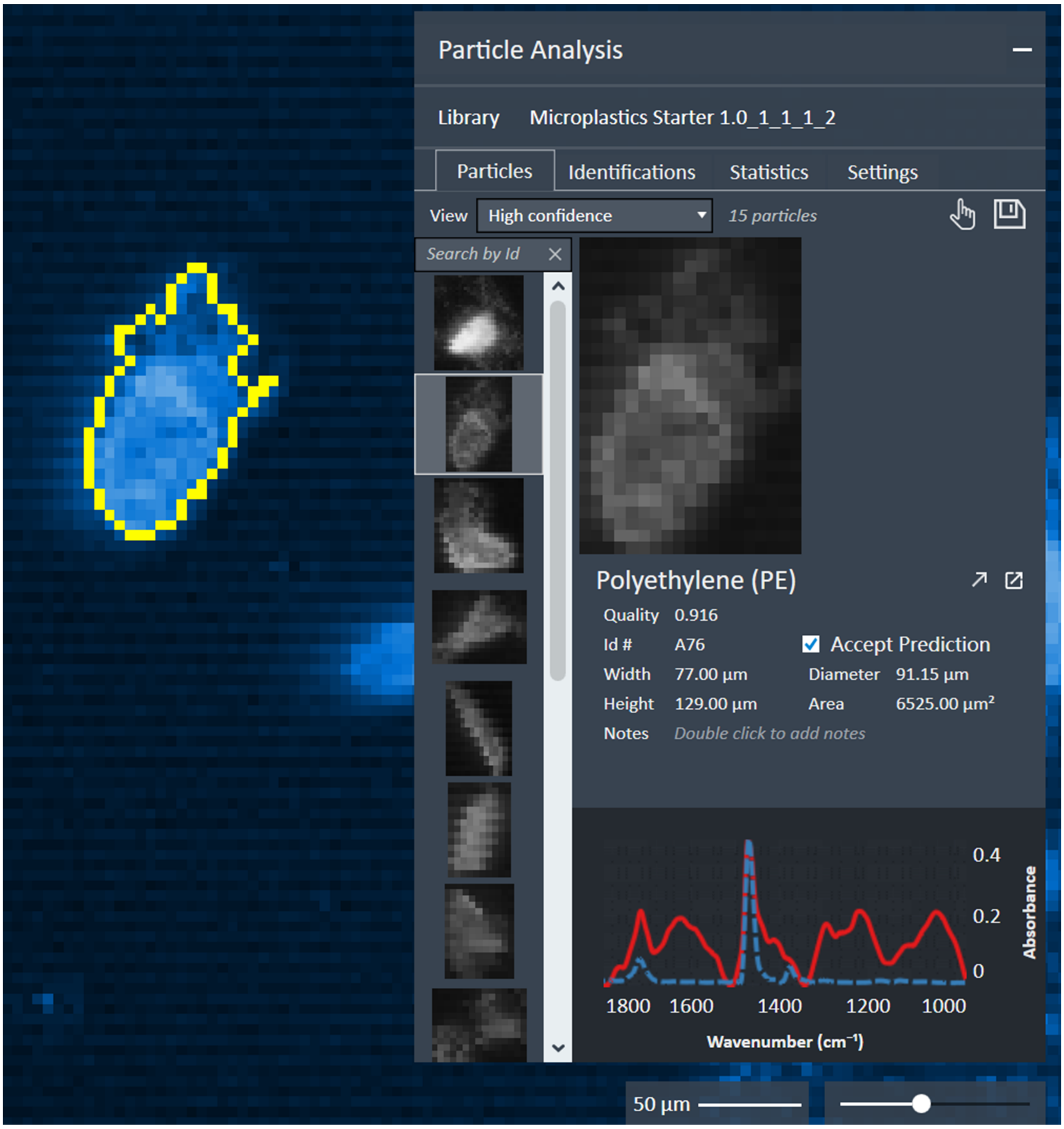

Supplement: Supplementary file 1 [file toxics-12-00761-s001.zip › toxics-3265493-supplementary.pdf]
